# Cerebrospinal fluid dynamics in idiopathic intracranial hypertension: a literature review and validation of contemporary findings

**DOI:** 10.1007/s00701-021-04940-x

**Published:** 2021-08-27

**Authors:** Aku L Kaipainen, Erik Martoma, Tero Puustinen, Joona Tervonen, Henna-Kaisa Jyrkkänen, Jussi J Paterno, Anna Kotkansalo, Susanna Rantala, Ulla Vanhanen, Ville Leinonen, Soili M Lehto, Matti Iso-Mustajärvi, Antti-Pekka Elomaa, Sara Qvarlander, Terhi J Huuskonen

**Affiliations:** 1 Neurosurgery KUH NeuroCenter, Kuopio University Hospital, and Faculty of Health Sciences, School of Medicine, Institute of Clinical Medicine, University of Eastern Finland, Kuopio, Finland; 2 Opthalmology KUH, Kuopio University Hospital, and Faculty of Health Sciences, School of Medicine, Institute of Clinical Medicine, University of Eastern Finland, Kuopio, Finland; 3 Division of Clinical Neurosciences, Department of Neurosurgery, Turku University Hospital, Turku, Finland; 4 Institute of Clinical Medicine, University of Oslo, Oslo, Norway; 5 R&D department, Division of Mental Health Services, Akershus University Hospital, Lørenskog, Norway; 6 Department of Psychiatry, University of Helsinki, Helsinki, Finland; 7 Department of Otorhinolaryngology, Kuopio University Hospital, Kuopio, Finland; 8 Eastern Finland Microsurgery Center, Kuopio University Hospital, Kuopio, Finland; 9 Department of Radiation Sciences, Radiation Physics, Biomedical Engineering, Umeå University, Umeå, Sweden; 10 Institute of Clinical Medicine / Neurology, University of Eastern Finland, Kuopio, Finland

**Keywords:** Idiopathic intracranial hypertension, Cerebrospinal fluid pressure measurement, Cerebrospinal fluid dynamics

## Abstract

**Background:**

Idiopathic intracranial hypertension (IIH) is a rare disease of unknown aetiology related possibly to disturbed cerebrospinal fluid (CSF) dynamics and characterised by elevated intracranial pressure (ICP) causing optic nerve atrophy if not timely treated. We studied CSF dynamics of the IIH patients based on the available literature and our well-defined cohort.

**Method:**

A literature review was performed from PubMed between 1980 and 2020 in compliance with the PRISMA guideline. Our study includes 59 patients with clinical, demographical, neuro-ophthalmological, radiological, outcome data, and lumbar CSF pressure measurements for suspicion of IIH; 39 patients had verified IIH while 20 patients did not according to Friedman’s criteria, hence referred to as symptomatic controls.

**Results:**

The literature review yielded 19 suitable studies; 452 IIH patients and 264 controls had undergone intraventricular or lumbar CSF pressure measurements. In our study, the mean CSF pressure, pulse amplitudes, power of respiratory waves (RESP), and the pressure constant (P_0_) were higher in IIH than symptomatic controls (*p* < 0.01). The mean CSF pressure was higher in IIH patients with psychiatric comorbidity than without (*p* < 0.05). In IIH patients without acetazolamide treatment, the RAP index and power of slow waves were also higher (*p* < 0.05). IIH patients with excess CSF around the optic nerves had lower relative pulse pressure coefficient (RPPC) and RESP than those without (*p* < 0.05).

**Conclusions:**

Our literature review revealed increased CSF pressure, resistance to CSF outflow and sagittal sinus pressure (SSP) as key findings in IIH. Our study confirmed significantly higher lumbar CSF pressure and increased CSF pressure waves and RAP index in IIH when excluding patients with acetazolamide treatment. In overall, the findings reflect decreased craniospinal compliance and potentially depleted cerebral autoregulation resulting from the increased CSF pressure in IIH. The increased slow waves in patients without acetazolamide may indicate issues in autoregulation, while increased P_0_ could reflect the increased SSP.

## Introduction

### Epidemiology of IIH

Idiopathic intracranial hypertension (IIH) is a disease with an incidence rate of 0.5–2.0/100,000/year [3, 31]. An IIH patient is typically a young obese woman with an increased intracranial pressure (ICP) but no established pathogenesis [31]. The main symptoms of IIH are headache and momentary visual symptoms, and if it is not properly diagnosed, treated, and followed up, it may lead to total loss of vision. The patients are described by unremarkable findings in (i) neurological examination, (ii) radiologic imaging and (iii) cerebrospinal fluids (CSF) composition. The first line of treatment for IIH is weight loss and medical treatment with acetazolamide. In selected cases, surgical interventions, such as shunting procedures, optic nerve sheath fenestration and sinus stenting may alleviate symptoms [3, 31] and in severely obese patients, gastric bypass surgery can be considered as a viable treatment for the IIH [3, 17, 31, 37]. IIH comorbidities, such as psychiatric disorders, are associated with worse subjective outcome of the treatments [40].

### Pathogenesis of IIH

The pathogenesis of IIH is unclear, but several theories attempt to describe why ICP increases among these patients. The majority of the IIH patients are young, obese and female, suggesting an essential role for neuroendocrinological dysfunction [31, 43, 44, 49]. Although altered metabolic, inflammatory and hormonal regulation may contribute to the development of the IIH [31], the dysfunction of CSF dynamics has been considered as the key element in understanding the aetiology of IIH [3, 7, 31, 45]*.* Deranged CSF dynamics and aquaporins could play a role in dysregulation of CSF production and in turn play a role in developing cerebral edema in IIH [3, 31, 43, 44].

### Disturbed cerebrospinal fluid dynamics in IIH

The proposed hydrodynamic mechanisms contributing to the development of the IIH include increased venous sinus pressure, CSF hypersecretion and CSF outflow obstruction. The CSF dynamics are based on three principal components: (i) production, (ii) circulation and (iii) absorption. The components can be measured with physiological infusion tests targeting the human CSF system.

The CSF infusion tests have been used to investigate mechanisms of IIH pathogenesis, but comprehensive reviews on their utilisation and findings in IIH patients are lacking [3, 31]. The extent of CSF hypersecretion in IIH patients has been investigated with initial infusion and magnetic resonance imaging (MRI) studies, but findings have been insignificant [31] and in general, objective evidence supporting increased cerebral blood volume or brain water content has not been strong [3, 31]. Some suggest that IIH is a result of increased venous pressure [3] and that increased sinus pressure caused by sinus stenosis is a key pathogenetic factor in selected cases. The sinus stenosis may also be secondary to the elevated intracranial pressure rather than the cause of the disease [31].

### Dynamic intracranial pressure analysis

Changes in CSF dynamics can be observed with continuous ICP, or lumbar CSF pressure, measurement when the CSF space is potentially infused or drained of liquids. Natural ICP changes during continuous measurements result from normal variations in cerebral blood volume (CBV), resulting in various ICP waveform components [22]. The most commonly studied long ICP waveforms are the slow waves or B-waves, lasting 30–120 s as first described by Lundberg [28]. The pulse amplitude (AMP) describes the magnitude of the cardiac-related waves and gives information about the dynamics of the cerebrospinal pressure, as it depends on both mean the ICP and craniospinal compliance [34].

Craniospinal compliance describes the relationship between changes in volume and pressure of the CSF system; this includes the intracranial compliance as well as the compliance of the spinal CSF compartment. Craniospinal (or intracranial) compliance is often illustrated by the exponential pressure/volume curve and can be quantified using the pressure volume index (PVI) [34]. When ICP increases the intracranial compliance (and thus also the craniospinal compliance) decreases, and the arterial pulses become more pronounced. The RAP index and the relative pulse pressure coefficient (RPPC) are both computational derivatives that provide information about the correlation between AMP and ICP. While the RPPC reflects the overall relationship and thus the pressure independent aspect of craniospinal compliance, the RAP index reflects whether the subject is presently in a high or low compliance state. Thus, the RAP index reflects present the location on the pressure/volume curve, while RPPC, as well as PVI, reflects the overall shape or steepness of this curve. Central venous pressure (CVP) may also affect the magnitude of continuous ICP measures. Respiratory waves are considered to reflect variations in the CVP and/or venous cerebral blood volume caused by e.g. changes in intrathoracic pressure. For example, in right atrial cardiac insufficiency, the CVP increases and the ICP waveform takes a more rounded shape [28].

### Aim of our study

This study clarifies the characteristics of CSF dynamics in IIH by reviewing the published studies utilizing CSF infusion tests in IIH patients. A literature review was performed on CSF dynamics and ICP waveform analytics in IIH according to Preferred Reporting Items for Systematic Reviews and Meta-Analyses (PRISMA) statement for reporting Systematic Reviews [36]. Based on a synthesis according to the literature, we established an evidence-based hypothesis for the altered CSF dynamics and aimed to validate some of the previous findings on a well-defined cohort. We conducted a retrospective analysis on verified IIH patients as compared to symptomatic controls. Common factors associated with IIH outcome, such as neuropsychiatric symptoms, were included in our analysis to evaluate whether their presence had previously unexplored effects on the lumbar CSF pressure measures.

## Materials and methods

### Literature research

**Fig. 1:**
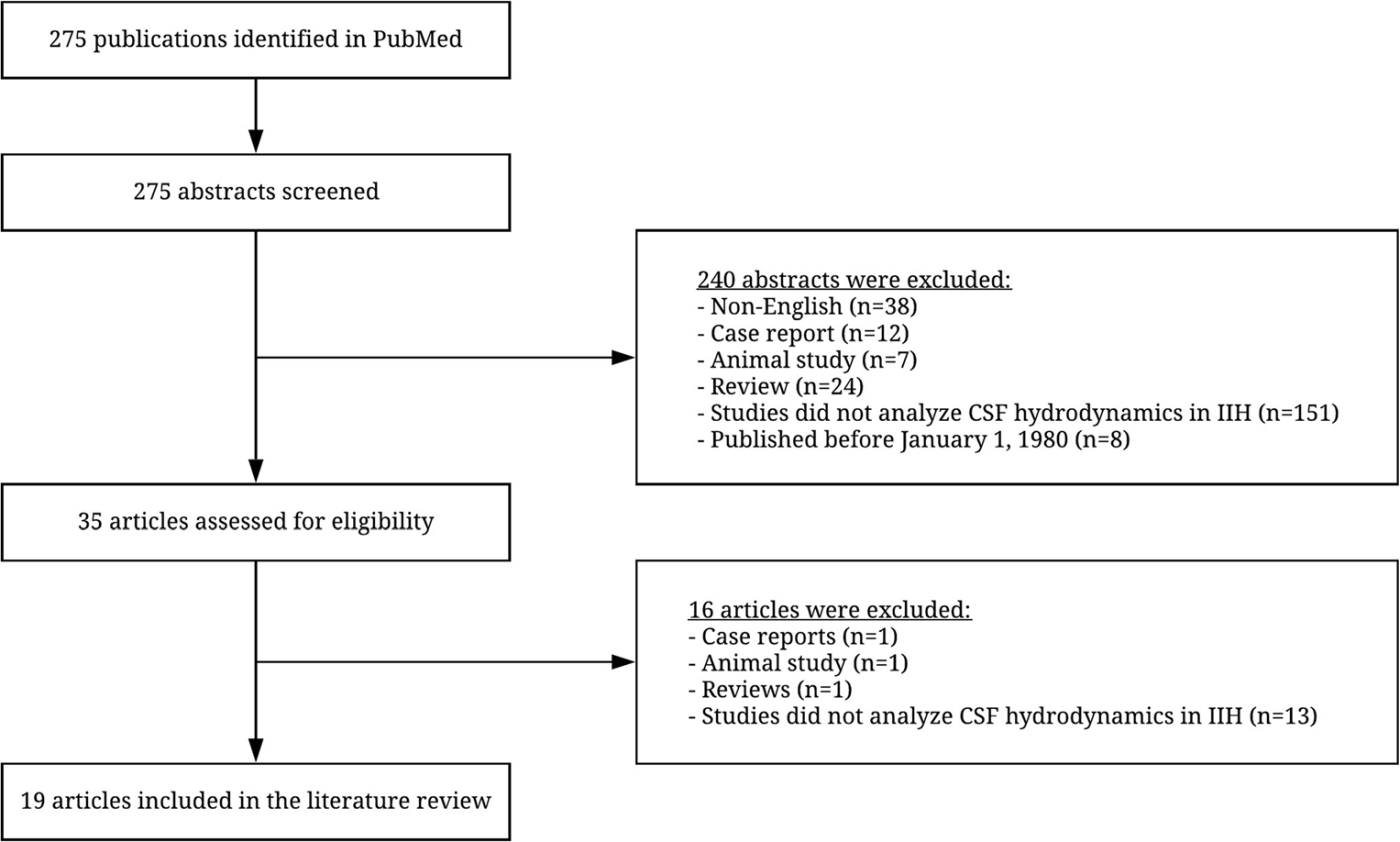
The flowchart of literature research

A database search from PubMed was conducted in accordance to the PRISMA guidelines [36] between January 1980 and December 2020 first using the following search terms: (“pseudotumor cerebri” OR “benign intracranial hypertension” OR “idiopathic intracranial hypertension” OR “IIH”) AND (“cerebrospinal fluid” OR “CSF”) AND (“absorption” OR “circulation” OR “dynamics” OR “homeostasis” OR “hydrodynamics” OR “infusion study” OR “secretion” OR “resorption”); and a second search with terms: (“pseudotumor cerebri” OR “benign intracranial hypertension” OR “idiopathic intracranial hypertension” OR “IIH”) AND (“intracranial pressure waveform” OR “lumbar infusion”). The search was limited to articles in English, and all case reports, reviews and animal studies were excluded (Fig. 1). All included studies are summarised in Table 1.

**Table 1:** Literature review of CSF dynamics in IIH

Authors	Data collection	Number of patients (% were females)	Mean age of patients (range)	CSF dynamics methods	Variables studied	CSF dynamics findings
Lalou et al. (2020)	Case series	*10 patients (90%)	41 (22–55)	*ICP monitoring (LIF) *DRCV intracranial	CSFP, SSP, sex, age	*CSFP and SSP are coupled *CSFP increase during CSF infusion produces an increase in SSP *During drainage, CSFP and SSP decrease until certain point, when CSFP may decrease further while SSP remains constant
Pradeep et al. (2020)	Prospective study	*13 patients (77%)	29.9 (16–40)	*ICP monitoring (LP)	OP, CP, V, age	*CSFP elevated
Markey et al. (2020)	A multicenter double-blind, placebo-controlled trial	*31 patients (100%)	31.2 (18–55)	*ICP monitoring (LP)	OP, age	*CSFP increased in IIH *11β-hydroxysteroid dehydrogenase type 1 inhibitor caused significant reduction in ICP
Lalou et al. (2020)	Retrospective cohort	*13 patients (85%) *5 patients not PTCS? (80%)	NA	*ICP monitoring (LIF)	CSFP, SSP, AMP, EL, RAP, LBP, UBP, sex	*CSFP and AMP elevated in IIH *SSP and EL were above threshold in IIH
Yilmaz et al. (2019)	Retrospective cohort	*29 IIH patients (93%) *30 controls (17%) *Mixed cohort	37.3 (18–57)	*ICP monitoring (LP)	OP, sex, age	*OP was increased in the presence of MR venography assessed transverse sinus compression
Griffith et al. (2018)	Retrospective cohort	*116 patients (94%)	33.5 (NA)	*ICP monitoring (LP)	OP, CP, V, EL, PVI	*As OP increases, the EL increases in a linear fashion while the PVI decreases
Chisholm et al. (2017)	Single-center retrospective cohort	*20 IIH patients (85%) *29 controls (62%) *Mixed cohort	35 (NA)	*ICP monitoring (LP)	OP, CP, V, EL, PVI	*Association between EL and PVI *EL is increased and PVI is decreased in IIH
Chari et al. (2017)	Retrospective review of prospectively collected cohort	*35 IIH patients (NA %) *41 Conservatively managed *Mixed cohort	34.9 (NA)	*ICP monitoring (intracranial)	CSFP, AMP, age	*ICP and AMP had a linear direct relationship in IIH *AMP and ICP were elevated in IIH
Thompson et al. (2017)	Retrospective cohort	*7 IIH patients (NA %) Mixed cohort	For whole cohort 45.8 (22–83)	*ICP monitoring (intracranial)	ICP, AMP	*A period of 24-h ICP monitoring is an accurate diagnostic option
Pickard et al. (2008)	Case series	*9 patients (89%)	41 (22–55)	*ICP monitoring (LIF)*DRCV (intracranial)	CSFP, SSP, sex, age	*Pcsf slightly exceeded Pss *CSF infusion provoked rises in Pcsf and Pss *CSF drainage decreased Pcsf compared to Pss
Karahalios et al. (1996)	Case series	10 patients (70%)	24 (2–40)	ICP monitoring (lumbar/intracranial)	CSFP, SSP, CVP, sex, age	*Pcsf elevated *CVP elevated *Pss elevated
Gideon et al. (1994)	Case-control study	*12 patients (67%) *10 controls	38 (12–61)	ICP monitoring (LIF)	ICP, R, sex, age	*Increased R *Increased CSF volume amplitude
Malm et al. (1992)	Prospective case-control study	*13 clear IIH patients (69%) *45 controls	35 (16–59)	ICP monitoring LIF)	CSFP, COP, F, PDOP, SSP	*CSFP increased *COP significantly reduced *PDOP increased *SSP was elevated
Hayashi et al. (1991)	Case series	*8 IIH patients (NA %) *Mixed cohort	NA	Continuous ICP monitoring (LIF and intracranial)	C, plateau waves (B and C waves), F	*C reduced
Lundar et al. (1990)	Case series	*6 patients (50%)	16 (3–39)	ICP monitoring (LIF and intracranial)	ICP, R, OP, PP, EPD, sex, age	*ICP increased *R from upper normal to pathologically increased *EPD labile but increased with a number of B waves
Shakhnovich et al. (1990)	Case-control study	*67 IIH patients (86%) *Mixed cohort	NA (14–63)	Continuous ICP monitoring (LIF)	EL, F, R, PIS	*EL increased *R significantly increased *No correlation between PIS and R
Borgesen et al. (1987)	Case-control study	*23 IIH patients (NA %) *Mixed cohort	NA	Continous ICP monitoring (intracranial)	ICP, R, F, duration of symptoms, Evans ratio, sex, age	*ICP increased with R *F decreased in the higher ICP values *The Evans ratio was higher in patients with low or normal ICP *Higher ICP in patients with a short duration of symptoms *R was lower in patients with a long duration of symptoms
Gjerris et al. (1985)	Case series	*14 patients (71%)	34 (12–61)	Continous ICP monitoring (intracranial)	ICP, C, CBF, plateau waves, B waves, sex, age	*ICP borderline elevated or increased *C decreased *All had B waves > 50 % of the time
Janny et al. (1981)	Case-control study	*16 patients (50%) *6 controls	25 (2–56)	Continuous ICP monitoring (intracranial)	ICP, CSFP, SSP, pressure gradient between CSFP-SSP, Evans ratio, R	*ICP was elevated *R was elevated

*AMP* pulse amplitude, *C* conductance of cerebrospinal fluid outflow, *CBF* cerebral blood flow, *COP* conductance of outflow pathways, *CP* closing pressure, *CSF* cerebrospinal fluid, *CSFP* cerebrospinal fluid pressure, *CVP* central venous pressure, *DRCV* direct retrograde cerebral venography, *EL* elasticity of the cerebrospinal fluid system, *EPD* epidural intracranial pressure, *F* cerebrospinal fluid formation rate, *ICP* intracranial pressure, *IIH* idiopathic intracranial hypertension, *LBP* lower breakpoint of the pulse amplitude, *LIF* lumbar infusion test, *LP* lumbar puncture, *NA* data not available, *OP* opening pressure, *PDOP* pressure difference across outflow pathways, *PIS* intrasinus pressure, *PP* plateau pressure, *PVI* pressure volume index, *R* cerebrospinal fluid outflow resistance, *RAP* index of cerebrospinal compensatory reserve, *SSP* sagittal sinus pressure, *UBP* upper breakpoint of the pulse amplitude, *V* volume of CSF removed

*Line break

### Study cohort

**Fig. 2:**
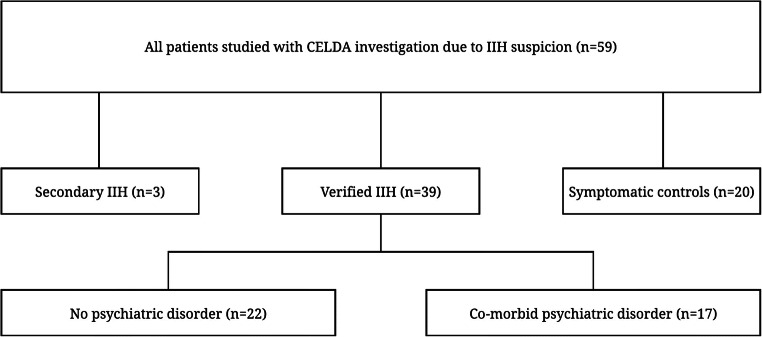
The flowchart of idiopathic intracranial hypertension (IIH) patients

This was a retrospective study on patients with International Statistical Classification of Diseases and Related Health Problems (ICD-10) G93.2 diagnosis, collected between January 1, 2000, and December 31, 2020, and formed a part of “Phenotype, Pathophysiology and Prognostic Factors of IIH” study, of which has been previously published a different analysis from the psychiatric comorbidity point of view [40]. Friedman criteria were applied to determine eligibility for inclusion in the cohort [13]. After the exclusion of (i) one subject due to poor CSF pressure measurement quality, (ii) three secondary IIH cases caused by venous sinus thrombosis, CNS infection and lithium medication and (iii) 20 suspected IIH cases that did not meet the criteria, we included 39 adult patients with verified IIH and a lumbar CSF pressure investigation using the CELDA™ System (Likvor AB, Umeå, Sweden) (Fig. 2). We included 39 patients with verified IIH diagnosis with lumbar CSF pressure measurement in our analysis; the demographic details are illustrated in Table 2. In total, 20 patients were studied with CELDA investigation due to suspicion of IIH, but did not fulfil the diagnostic criteria of IIH: these patients were referred as symptomatic controls.

**Table 2:** IIH patients compared to symptomatic controls

	Verified IIH	Symptomatic controls	
	*n* = 39	*n* = 20	*p*
Females	35 (89.7%)	18 (90.0%)	0.975
Mean age in years	30.2 ± 11.0	29 ± 11.6	0.808
Mean BMI (kg/m^2)^	36.0 ± 6.5	28.7 ± 6.5	0.001*
Mean OP (mmHg)	29.5 ± 7.7	18.5 ± 9.7	< 0.001*
Mean CSF protein count (mg/l)	299.2 ± 142.9	252.1 ± 70.9	0.247
Presenting symptoms			
Headache	31 (79.5%)	18 (90.0%)	0.308
All visual symptoms	33 (84.6%)	13 (65.0%)	0.085
Tinnitus	8 (20.5%)	4 (20.0%)	0.963
Dizziness	5 (12.8%)	1 (5.0%)	0.347
Neuro-opthalmological findings at diagnosis			
Optic nerve edema			
Slight	8 (20.5%)	7 (35.0%)	< 0.001*
Unilateral	5 (12.8%)	1 (5.0%)	
Bilateral	26 (66.7%)	3 (15.0%)	
Visual field defects	20 (51.3%)	0 (0.0%)	0.001*
Mean visual acuity			
Right eye	1.05±0.31	1.18±0.23	0.133
Left eye	1.09±0.33	1.18±0.28	0.332
Neuroradiological findings at diagnosis			
Presence of empty sella			
No	15 (42.9%)	11 (61.1%)	0.208
Yes	20 (57.1%)	7 (38.9%)	
Flattened sclera	7 (20.6%)	2 (11.1%)	0.390
Increased CSF around optic nerve	16 (47.1%)	5 (27.8%)	0.382
Intraocular protrusion of optic nerve head	2 (5.9%)	1 (5.6%)	0.962
Increased tortuosity of optic nerve	4 (11.8%)	1 (5.6%)	0.470
Neuropsychiatric findings at diagnosis			
Diagnosis of psychiatric disorder (ICD-10)			
None	22 (56.4%)	15 (75.0%)	0.431
Bipolar disorder, type II (F31.8)	2 (5.1%)	0 (0.0%)	
Major depressive disorder (F33)	14 (35.9%)	5 (25.0%)	
Dissociation disorder (F44.9)	1 (2.6%)	0 (0.0%)	

*p* Values of categorical variables from χ^2^ test and *p* values of continuous variables for ANOVA

*BMI* body mass index, *CSF* cerebrospinal fluid, *OP* opening pressure

### Clinical and treatment variables

Clinical data on treatment periods and follow-up visits from referring hospitals and Kuopio University Hospital (KUH) have been included into the Kuopio IIH database. Relevant medical charts, radiological imaging, laboratory results and clinical follow-up evaluations were analysed up until June 2020.

Before diagnosis of IIH:

- History of psychiatric diseases (diagnoses and psychiatric medications).

Baseline at diagnosis of IIH:

- Demographics [sex, age, body mass index (BMI), symptoms and CSF opening pressure (OP)];
- Neuro-ophthalmological findings (visual acuity, papillae and visual fields);
- Magnetic resonance imaging (MRI) findings evaluated by a neuroradiologist;
- Lumbar CSF pressure parameters determined with a CELDA CSF infusion apparatus.

Follow-up during treatment of IIH:

- Treatment types: (a) conservative (weight loss, medication), (b) surgical treatments (CSF diversion, gastric by-pass), (c) combined;
- Treatment outcomes after (a) medical, (b) both medical and surgical treatments combined;
- Neuro-ophthalmological outcome: (a) no papilledema, (b) partial resolution, (c) no improvement;
- Symptomatic outcome: (a) symptomless, (b) partial recovery, i.e. ongoing symptoms such as headache, tinnitus, fatigue, dizziness, balance problems and need for continuing medical treatment, (c) no improvement.

### Lumbar CSF pressure measurement and analysis

**Fig. 3:**
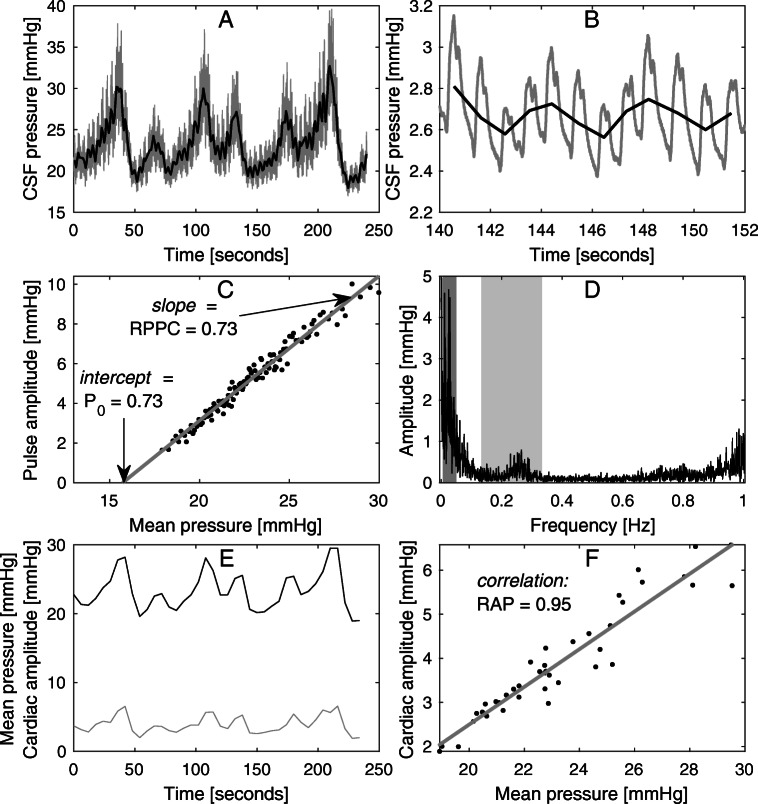
Illustration of lumbar CSF pressure parameters. **A** Four minutes of the pressure measurement for a verified IIH case, with CSF pressure at 100 Hz in grey and pressure averaged over each cardiac cycle in black, illustrating slow wave activity. **B** A 12-s segment of the measurement, illustration cardiac-related pulse waves and respiratory waves. **C** Linear regression (grey line) of mean pressure and pulse amplitude data from the entire measurement (black dots), where RPPC is the slope of line and P_0_ is the intercept with the pressure axis (at ~ 16 mmHg). **D** Frequency spectrum for the same measurement, with the frequency window for slow waves shaded in dark grey and the window for respiratory waves shaded in light grey (the cardiac peak can be seen at the right edge of the graph, at around 1 Hz). **E** Mean pressure and cardiac amplitude (determined by frequency analysis) for each 6 second window of the 4 min, corresponding to the data used to determine one estimate of RAP index. **F** The cardiac amplitude data from panel E plotted against the corresponding mean pressure values; the linear correlation between them provides the RAP index estimate

The subjects underwent a 20- to 30-min-long continuous CSF pressure measurement in the supine position, using a CELDA™ infusion apparatus (Likvor AB, Umeå, Sweden), where contact with the CSF space was made via lumbar puncture. The raw pressure data, with a sampling frequency of 100 Hz, was saved for each measurement, and this data was retrospectively re-analysed in the present study. All lumbar CSF pressure parameters were averaged from 10 to 20 min of measurement, always after an initial 10-min period where the lumbar CSF pressure was allowed to stabilise after the lumbar puncture. This stabilisation period, as well as the patient being in supine position, contributes to a well-controlled assessment of lumbar CSF pressure, which has previously been shown to agree well with ICP as long as there is a communicating CSF system [26], which was the case for all our subjects. Thus, even in terms of the CSF pressure level, these measurements offer more information than the momentary measure of an “opening pressure”. An illustration of the CSF pressure measurement and corresponding parameters for one subject is shown in Fig. 3. Lumbar CSF pressure was defined as the mean CSF pressure over this measurement period and AMP as the mean of the pulse amplitudes as assessed in the time domain using an algorithm that was in-house developed at Umeå university [41]. The average values of lumbar CSF pressure and pulse amplitude for each consecutive period of ten cardiac cycles, as determined with this algorithm, were also used to assess the linear relationship between lumbar CSF pressure and pulse amplitude for each patient. As illustrated in Fig. 3C, the slope of this relationship is called RPPC [25] and the intercept with the pressure axis corresponds to P_0_, a constant in the mathematical model describing CSF dynamics [1]. This linear relationship is only valid in a certain, individual pressure range, below which the relationship is constant [46]. Cases where the value of RPPC was below 0.1 or P_0_ was below zero were interpreted as the measurement occurring, at least for a large proportion of the time, in the lower pressure range where the linear relationship is not valid, and these values of RPPC and P_0_ were thus discarded.

Additionally, the power of slow (SLOW) and respiratory waves (RESP) were assessed by frequency domain analysis [10]. The total power of the relevant frequency range (in mean corrected data) was determined and converted to an equivalent amplitude. The calculated value can thus be interpreted as what the amplitude would be for a single sine wave, continuous thorough the measurement period, if it carried the same power as the total slow wave or respiratory activity. The frequency range was set to 0.0055–0.05 Hz for slow waves and to 0.1333–0.3333 Hz for respiratory waves (8–20 respiratory cycles per minute) (see Fig. 3D). The RAP index was determined as the correlation coefficient between mean lumbar CSF pressure and cardiac amplitude assessed from the frequency domain according to recommendations in the literature [10]. In brief, the cardiac amplitude is determined from the frequency spectrum peak at the (fundamental) cardiac frequency for each 6-s time window, along with a corresponding mean pressure value. The linear correlation coefficient is then calculated for a sliding window of 40 pairs of values (see Fig. 3E), resulting in an estimate of RAP index for a period 4 min (which is updated for every 6-s period). Mean RAP index for the entire measurement period was calculated as the final value of RAP index for each subject.

### Statistical analysis

Patient demographics, IIH-specific details, neuro-ophthalmological findings, neuroradiological findings and outcome data were analysed with ANOVA for continuous variables and Pearson’s χ^2^ analysis for categorical variables. The statistical analyses were performed with SPSS (IBM, v. 24.0). A *p* value of < 0.05 was considered as statistically significant. For the lumbar CSF pressure measurements, all statistics were calculated using the PASW Statistics module (v. 18.0). Normality of the distributions of the continuous parameters was assessed with Shapiro–Wilks and Lilliefors tests. Since several of these parameters were not normally distributed in at least one of the investigated groups (see Tables 1 and 2), all comparisons between groups were performed using Mann–Whitney *U* tests and correlations were assessed using rank correlation (Spearman’s rho). For statistical analyses, outcome variable was pooled so that no improvement or slight improvement with persisting symptoms were considered as unfavourable outcome and complete resolution of symptoms as favourable outcome despite some patients had still medication on use.

### Ethical standards

Approval for this study was received from the Ethics Committee of the Kuopio University Hospital (284/2016). The study was conducted in accordance with the principles of the Declaration of the Helsinki and “The Strengthening the Reporting of Observational Studies in Epidemiology” (STROBE) guidelines were used in reporting our findings. All patients gave their informed consent for this study.

## Results

### Literature review of CSF dynamic findings in IIH

In our literature review, we found 452 IIH patients who were included in 19 studies with CSF dynamics measures summarised in Table 1. Majority of the studies were done in the end of twentieth century as well as in the end of the previous decade. Cohorts were mainly rather small (range 6–116 IIH patients) [5, 8, 9, 14–16, 19–21, 23, 24, 27, 29, 30, 39, 42, 48, 51]. Most of the studies were case series, case-control studies and retrospective cohorts [5, 8, 9, 14–16, 19, 20, 23, 24, 27, 29, 38, 42, 48]. Three of the studies were prospective [29, 30, 51]. The IIH patients were relatively young (range 2–63 years) and majority were female, which supports earlier knowledge.

All studies measured CSF pressure invasively either intracranially [5, 8, 15, 20, 48] or via a lumbar route with infusion test [14, 24, 29, 42] or without [9, 16, 30, 39, 51] or both intracranially and via a lumbar route [19, 21, 23, 27, 38]. Studies reported CSF dynamics such as CSF opening pressure, CSF formation rate, resistance/conductance of CSF outflow, continuous pressure values, and sagittal sinus pressure (SSP) [5, 8, 9, 14–16, 19–21, 23, 24, 27, 29, 30, 38, 39, 42, 48, 51]. All main findings from our literature review are summarised in Table 1.

### Characteristics of verified IIH patients and symptomatic controls at diagnosis

Patients with verified IIH had a mean follow-up time of 41.2 (SD 43.9) months, and control patients were followed for s23.3 (SD 16.2) months. The gender distribution, age and the CSF protein count at diagnosis were equal for both groups. Also, the presenting symptoms were similar; most commonly the subjects presented with headache and visual disturbances. Neuroradiological findings did not differ between the IIH patients and symptomatic controls; the most common findings were empty sella and excess CSF around the optic nerve. A total of 15 (45.7%) patients with IIH had pre-existing psychiatric diagnoses. For the verified IIH patients, the most common psychiatric diagnosis was major depressive disorder (MDD), which was found in 14 (35.9%) patients. Bipolar disorder was found in two (5.1%) patients, and one patient had a dissociative disorder (2.6%). In the symptomatic control group, five (25%) had MDD at presentation, while the rest did not have any psychiatric comorbidity in comparison with verified IIH patients (*p* = 0.431). We found no differences in the baseline characteristics between verified IIH patients with or without psychiatric comorbidity (Table 2).

The mean of first opening CSF pressure was significantly higher [29.5 (SD 5.5) mmHg] for the verified IIH patients, as compared with the symptomatic controls [18.5 (SD 9.7) mmHg] (*p* < 0.001). In addition, the verified IIH patients had higher mean BMI at diagnosis 36.0 (SD 6.5) kg/m^2^ as compared with the symptomatic controls [28.7 (SD 6.5) kg/m^2^] (*p* = 0.001). Verified IIH patients had more often severe optic nerve findings as compared with the symptomatic controls. Twenty (51.3%) IIH patients had visual field defects at diagnosis whereas no patient from the symptomatic control group had a visual field defect (*p* = 0.001). However, there was no difference in visual acuity at presentation (Table 2).

### CSF pressure measurements in IIH patients and symptomatic controls

The lumbar CSF pressure parameters from the CELDA measurements are presented in Table 3. When comparing IIH cases versus symptomatic controls, all parameters differed significantly between the groups (*p* < 0.01) except the RAP index, SLOW and RPPC. Fifty percent of the IIH cases and 25% of the controls were taking acetazolamide at the time of the pressure measurement (*p* = 0.068). To ensure that this did not skew the comparison between the groups, a corresponding analysis was also performed for only the cases without acetazolamide treatment. For these 33 subjects (18 with verified IIH), the identified differences between the groups were similar to the original analysis, except that the RAP index and SLOW were also significantly higher in the IIH cases (RAP index: median (IQR) 0.86(0.13) vs 0.59(0.45), *p* = 0.017; SLOW: median (IQR) 1.1(1.2) mmHg vs 0.7(1.0) mmHg, *p* = 0.025).

**Table 3:** Lumbar CSF pressure parameters for verified IIH cases and symptomatic controls

	Verified IIH cases	Symptomatic controls	
Lumbar CSF pressure parameter	Median (IQR)	Median (IQR)	*p*
Mean CSF pressure (mmHg)	20.7 (7.75)	13.0 (6.5)	**< 0.001**
AMP (mmHg)	4.5 (6.0)	1.5 (2.6)	**< 0.001**
SLOW (mmHg)	1.0 (1.1)	0.6 (0.9)	0.109
RESP (mmHg)	0.8 (0.5)	0.3 (0.2)	**< 0.001**
RAP index	0.82 (0.29)	0.63 (0.58)	0.288
RPPC^1^	0.52 (0.26)	0.40 (0.26)	0.228
P_0_ (mmHg)^1^	10.8 (4.4)	7.3 (4.6)	**0.008**

All *p* values from Mann–Whitney *U* tests comparing the two groups

Bold values denote statistical significance at the *p* < 0.05 level

*IQR* interquartile range

†Not normally distributed

^1^*N* = 30 IIH cases, *N* = 11 controls

### CSF pressure measurements in verified IIH patients

Only the power of respiratory waves showed significant positive correlation to BMI (RESP: rho = 0.37, *p* = 0.033). None of the pressure parameters showed significant rank correlation to levels of CSF protein (*p* > 0.2). When analysing only the IIH subjects that were not taking acetazolamide at the time of measurement, the correlation between RESP and BMI was stronger (rho = 0.76, *p* = 0.001), but no other trends were identified. In subjects with excess CSF around the optic nerve the RPPC [(*N* = 13 vs 13, median (IQR) 0.41(0.28) vs 0.62(0.27), *p* = 0.009] and RESP [*N* = 16 vs 17, median (IQR) 0.6(0.5) mmHg vs 0.9(0.6) mmHg, *p* = 0.008)] were significantly lower than in those without, respectively. RPPC was also lower in IIH subjects with empty sella compared to those without (*N* = 15 vs 15, median (IQR) 0.45(0.14) vs 0.63(0.28), *p* = 0.041). Comparisons between IIH subjects with or without psychiatric comorbidity (*N* = 21 vs 17) showed higher lumbar CSF pressure (median (IQR) 22.7(7.0) mmHg vs 17.6(8.3) mmHg, *p* = 0.038), as well as trends toward higher RESP (median (IQR) 0.9(0.6) mmHg vs 0.7(0.6) mmHg, *p* = 0.083), and lower RPPC (*N* = 15 vs 15, median (IQR) 0.45(0.20) vs 0.61(0.26), *p* = 0.061) for subjects with comorbidity than those without (all other parameters: *p* > 0.1).

### Treatment modalities and outcome of IIH patients

A total of 21 (53.8%) IIH patients were prescribed acetazolamide medication during lumbar CSF pressure measurement due to debilitating symptoms and assessment for possible CSF diversion. A total of 18 (46.2%) patients had no medication or it had been started within two weeks prior to CELDA investigation. The acetazolamide treatment was reported beneficial, i.e. complete resolution of all symptoms was observed in 17 (48.6%) of all patients, while partial recovery only or no improvement was noted in 18 (51.4%) patients at the end of follow-up (Table 4). A CSF diversion by lumboperitoneal shunting was conducted in 9 (23.1%) patients. Gastric bypass surgery was done in two (5.1%) patients. After conservative and operative treatments, 21 (60 %) patients reported themselves to be symptomless, hence having favourable outcome. The IIH patients with psychiatric comorbidity had significantly worse outcome as compared to patients without such history (37.5% vs. 78.9%) (*p* = 0.013) (Table 4).

**Table 4:** Outcome for IIH patients after treatment

	IIH patients with psychiatric comorbidity	IIH patients without psychiatric comorbidity	*p*
Neuro-ophthalmological outcome after all treatments			
No papillaedema	8 (50.0%)	15 (78.9%)	0.072
Papillar atrophy or partial resolution of papillaedema	8 (50.0%)	4 (21.1%)	
Effect of acetaloamide on papillae and symptoms			
Complete resolution of symptoms (with or without medication)	5 (31.3%)	12 (63.2%)	0.060
Slight or no improvement	11 (68.8 %)	7 (36.8%)	
Effect of conservative and surgical treatments combined on papillae and symptoms			
Complete resolution of symptoms	6 (37.5%)	15 (78.9 %)	**0.013**
Slight improvement	10 (62.5%)	4 (21.1%)	

All *p* values from χ^2^ test

Bold values denote statistical significance at the *p* < 0.05 level

The visual acuity at diagnosis was better in the IIH patients with favourable outcome as compared with IIH patients with unfavourable outcome (*p* = 0.018 right eye and *p* = 0.001 left eye). Neuro-ophthalmological outcome was assessed as a degree of resolution of papilledema, and up to 65.7% of all patients had physiological papillae at the end of follow-up, and partial resolution was noted in 28.6% and 5.7% had papilla atrophy at the end of follow-up (Table 4).

For the IIH cases, values of the lumbar CSF pressure parameters grouped according to outcome (favourable versus unfavourable) are presented in Table 5. The ratio of subjects using acetazolamide at the time of the measurement was similar (55% vs. 54%, chi-square test *p* = 0.61), but none of the pressure parameters differed significantly between these groups (*p* > 0.2). There were no differences in lumbar CSF pressure parameters between IIH patients with complete resolution of papilledema and those with partial or no resolution (*N* = 23 vs 12, *p* > 0.3). When comparing results from IIH subjects who were not taking acetazolamide at the CSF pressure measurement to those who were (*N* = 18 vs 17), there were trends towards higher lumbar CSF pressure [median (IQR) 22.9 (7.3) mmHg vs 17.9 (9.0) mmHg, *p* = 0.051], increased RAP index [median (IQR) 0.86 (0.13) mmHg vs 0.67 (0.48) mmHg, *p* = 0.069] and increased power of slow waves [median (IQR) 1.1 (1.2) mmHg vs 0.9 (0.8) mmHg, *p* = 0.086] in patients without acetazolamide treatment.

**Table 5:** Lumbar CSF pressure parameters for verified IIH cases with favourable or unfavourable outcome

	Favourable outcome (*N* = 21)	Unfavourable outcome (*N* = 14)	
Lumbar CSF pressure parameter	Median (IQR)	Median (IQR)	*p*
Mean CSF pressure (mmHg)	20.7 (8.0)	18.7 (9.3)	0.419
AMP (mmHg)	4.6 (5.6)	3.0 (7.6)	0.973
SLOW (mmHg)	1.0 (1.0)	1.0 (1.1)	0.727
RESP (mmHg)	0.9 (0.7)	0.8 (0.4)	0.987
RAP index	0.82 (0.27)	0.79 (0.48)	0.219
RPPC^1^	0.53 (0.33)	0.47 (0.22)	0.366
P_0_ (mmHg)^1^	9.6 (3.5)	11.7 (5.1)	0.228

All *p* values from Mann–Whitney *U* tests comparing the two groups

*IQR* interquartile range

*Not normally distributed

^1^*N* = 17 favourable outcome, *N* = 10 unfavourable outcome

## Discussion

In this study, we conducted a literature review of CSF dynamics in IIH and investigated different lumbar CSF pressure parameters in a well-described retrospective cohort. The systematic review of the literature mainly revealed consistently increased CSF pressure and increased resistance to CSF outflow. Our analysis of the retrospective cohort showed that mean lumbar CSF pressure and BMI were higher in verified IIH patients compared to symptomatic controls, and that both fast and slow dynamic changes in lumbar CSF pressure were increased. We confirmed that psychiatric comorbidity is common in IIH and found that lumbar CSF pressure was more often higher in cases with such comorbidity. We observed that the use of acetazolamide may have obscured an increase in RAP and SLOW measures in true IIH patients.

### Literature review of CSF dynamic findings in IIH

In our literature review of CSF dynamic findings in IIH, we found that lumbar CSF pressure or ICP was elevated in IIH patients [5, 8, 9, 15, 16, 19–21, 23, 24, 27, 29, 30, 38, 39, 48, 51], and the resistance to CSF outflow increased [5, 14, 20, 27, 42]. CSF pressure and AMP were associated with linear direct relationship [8]. Also, a positive linear relationship between ICP and resistance of CSF outflow has been identified [5]. Moreover, elevated SSP is a common finding in IIH patients [4, 21, 24, 29, 38, 47], although only recently prospective attempts have been made to verify the range of normal variation in non-IIH populations (ClinicalTrials.gov ID: NCT03948971). CSF pressure and SSP appears to be coupled in IIH and when CSF pressure increases during CSF infusion, it produces an increase in SSP [38] and its vasogenic components [23]. During drainage, both pressures decrease until a certain point, when CSF pressure may decrease further, while SSP remains constant [23, 38]. Opening pressure appears to be increased in the presence of MR venography assessed transverse sinus compression [51]. In one study, CSF formation rate tended to decrease on IIH patients with higher than average ICP values [5]. One study illustrated the so-called B waves during the continuous CSF pressure monitoring, present for at least 50% of the time [15]. The craniospinal elastance appears to be higher in IIH patients [9, 16, 24, 42], corresponding to a reduced craniospinal compliance, since this is the inverse of elastance. PVI represents the calculated volume required to raise the CSF pressure (or ICP) by a factor of 10 and describes the relationship between pressure and compliance [32]. One previous study also found that PVI was decreased in IIH [9], which could suggest that the observed changes in craniospinal elastance or compliance are not only secondary to an increased pressure, but also reflect a change in the overall pressure/volume curve. That study also investigated the relationship between lumbar CSF opening pressure and PVI, and found no dependency [9]. However, another study found a negative linear relationship between lumbar CSF opening pressure and PVI in IIH [16], which could complicate the comparison of PVI between IIH and other groups. Recent studies thus suggest that the craniospinal compliance dynamics may differ in patients with IIH, which could provide insight into the disease pathogenesis. However, further studies confirming these results and comparing similar measurements in patients with and without IIH are needed to determine whether potential differences in craniospinal compliance in IIH reflect expected variations due to the changes in pressure or may be related to pathophysiologic changes in IIH [16]. Additionally, all the reported findings regarding compliance or elastance were based on measurements of lumbar CSF pressure, rather than intracranial pressure. It should be noted that the observations are of changes in craniospinal compliance, which includes the compliance of both the intracranial and spinal CSF compartments, and thus it is not possible to draw distinct conclusions about changes in the intracranial compliance. When these compartments are communicating, as is generally the case in IIH, measurements in the two compartments should be closely related, but it could be of interest to confirm these findings with intracranial measurements.

Increased venous sinus pressure has been regarded as one of the possible causes of IIH. In addition, changes of the cerebral cortical capillaries and blood–brain barrier dysfunction are associated with evolvement of IIH [12, 18]. Classically, it is thought that the majority of the drainage of CSF into the venous compartment takes place through the arachnoid granulations that penetrate the sagittal sinus but also alternative routes have been described [11]. Recently found cerebral lymphatic system has been thought to also support the CSF outflow [31]. Thus, an increased resistance to CSF outflow in IIH could be indicative of a disturbance in any of these suggested absorption routes. Furthermore, the rate of absorption of CSF depends on the pressure gradient between the subarachnoid space and venous sinus, hence with an increase in venous pressure a concomitant increase in CSF pressure is needed to maintain absorption rates [11]. Because of this relationship between absorption and venous pressure, the observed coupling of SSP and CSF pressure during infusion in IIH [23, 38] could affect the measurement of resistance to CSF outflow in IIH subjects, since this measurement depends on an assumption of a stable SSP during the infusion. If SSP rises during the infusion the estimated resistance could be falsely high, thus, in order to confirm an increased resistance in IIH infusion investigations should be performed with simultaneous measurement of SSP.

### Retrospective cohort analysis: baseline patient characteristics and lumbar CSF pressure parameters

The mean age and CSF protein count at diagnosis was similar in both groups. The presenting symptoms were similar and most commonly these patients presented with headache and visual disturbances. Neuroradiological findings did not differ between verified IIH patients and symptomatic controls, most commonly these patients presented with empty sella or increased CSF around the optic nerve. However, the mean measured opening pressure and BMI were significantly higher for the verified IIH patients, as compared with the symptomatic controls, as expected. In addition, the true IIH patients more often had severe optic nerve findings and visual field defects at diagnosis, but there was no difference in visual acuity at presentation.

When comparing verified IIH cases versus symptomatic controls, all lumbar CSF pressure parameters differed significantly between the groups except RAP index, power of slow waves and RPPC. When all cases who were on acetazolamide during the pressure measurement were excluded, the RAP index and slow waves were also significantly higher in IIH patients than controls. Increased fast and slow waves in lumbar CSF pressure (AMP, RESP, SLOW) as well as increased RAP index likely reflect reduced craniospinal compliance in IIH, as would be expected at the increased lumbar CSF pressure level. These results are not entirely unexpected but have not been previously established in the literature. Amplitudes of CSF pressure waves, specifically pulse amplitudes, measured via the lumbar route, with the methodology used in this study, are typically somewhat smaller than if measured intracranially, though there is a very strong correlation[2]. This is because the pressure waves, which originate intracranially, are dampened somewhat as they are transmitted along the spinal compartment. Thus, the amplitude levels measured here are not directly comparable to intracranial amplitude levels. While the significant differences between the two groups in our cohort likely reflect a genuine difference in intracranial amplitudes as well, the magnitude of that difference may be not be the same, since the dampening may depend slightly on the pressure level[2]. The observed increase of P_0_ in IIH patients compared to symptomatic controls could support the previous finding of increased sagittal sinus pressure in IIH [21, 29, 38], since this pressure constant has been suggested to depend on venous pressure[1] — and at least venous flow [6]. According to the mathematical model of CSF dynamics, P_0_ sets the “baseline” for the lumbar CSF pressure level, i.e. a change in P_0_ results in an equal change in (mean) lumbar CSF pressure. In terms of the classical exponential pressure/volume curve for CSF pressure, a change in P_0_ corresponds to moving the entire curve along the pressure axis, rather than moving the state of the patient along the curve. Accordingly, craniospinal compliance decreases as the difference between lumbar CSF pressure and P_0_ increases, i.e. an increased lumbar CSF pressure would not result in reduced craniospinal compliance if P_0_ increased in parallel. Based on our results, the increase in P_0_ in IIH was less than half of the increase in lumbar CSF pressure (Table 3), which explains how we also found evidence of decreased craniospinal compliance. Thus, in IIH, there may be an effect on both the baseline of lumbar CSF pressure and on the volume-related aspect of lumbar CSF pressure, i.e. a shift of both the pressure/volume curve and of the state of the patient along the curve. Additionally, as revealed in the literature review, there may be an effect on the steepness of the pressure/volume curve, as indicted by a reduction of PVI [9], which we did not measure in our cohort. PVI is closely related to RPPC, which we did measure and found not to be altered in IIH, but RPPC also depends on the change of cerebral arterial blood volume with each heartbeat, which could be a confounder in this comparison. Also, we were not able to assess RPPC in all subjects, because we assessed this parameter based on natural pressure variability rather than with infusion tests.

The increased value of SLOW we found in the IIH cases without acetazolamide treatment could stem from a decreased craniospinal compliance, resulting in higher amplitudes, but it could also mean that SLOW waves appear more often in IIH. The power of slow waves reflects both the degree of occurrence and the amplitude of the waves, i.e. the power can increase if waves occur during a higher proportion of time or if the amplitude of the waves increase. Since slow waves are expected to appear in response to variations in intracranial blood volume or blood pressure, this could potentially indicate an issue with cerebral autoregulation in IIH. However, without simultaneous measurement of arterial blood pressure, the origin of the waves cannot be confirmed, and thus, autoregulation was not specifically assessed. Also, with the relatively short duration of measurement of this study, i.e. < 1 h, it was not possible to reliably determine if there is more slow wave activity in IIH in general, though such results would be in line with Gjerris et al [15]. These results warrant studies on other indicators of cerebral autoregulation in future IIH research.

In IIH patients, there was significant correlation between power of respiratory waves and BMI, but not between any other lumbar CSF pressure parameters and BMI or between lumbar CSF pressure parameters and CSF protein levels. When analysing only the IIH subjects that were not using acetazolamide at the measurement occasion, the correlation between RESP and BMI increased further. The respiratory waves reflect variations in venous cerebral blood volume and/or CVP, so this observation could relate to increased venous pressure or increased respiratory variation in venous pressure in subjects with high BMI.

Comparing lumbar CSF pressure parameters in IIH cases with and without empty sella turcica did not reveal any differences. In IIH subjects with excess CSF around the optic nerve, RPPC and power of respiratory waves were significantly lower than for subjects without this radiological finding. The implications of these results are unclear; though a reduced RPPC may be indicative of a worsened pressure-independent component of craniospinal compliance, i.e. the aspect of compliance that defines the steepness of the pressure/volume curve, which is often described by the PVI [33].

### Lumbar CSF pressure and outcome in IIH patients

Only about half of the IIH patients benefitted from acetazolamide treatment, and further surgical intervention was required for eleven patients. After conservative and operative treatments, the overt outcome improved slightly as 60% of patients reported themselves to be symptomless, hence having favourable outcome. The visual acuity at diagnosis was better for the IIH patients with favourable outcome as compared with IIH patients with unfavourable outcome. This finding might suggest that if the IIH is diagnosed early and the treatment has been started promptly, the IIH-related symptoms have progressed less leading to better outcome. No lumbar CSF pressure parameters differed significantly between these groups. Neuro-ophthalmological outcome was assessed as a degree of resolution of papilledema, and up to 65.7% of all patients had physiological papillae at the end of follow-up, there were no differences in lumbar CSF pressure parameters when stratified by neuro-ophthalmological outcome.

When comparing results from IIH subjects who were taking acetazolamide to those who were not, there were trends toward higher lumbar CSF pressure, power of slow waves (SLOW) and RAP index in subjects without acetazolamide. While not statistically significant, the trends for RAP index and SLOW motivate the post hoc analysis comparing only verified IIH cases and symptomatic controls without acetazolamide treatment, where these two parameters showed significant differences between the groups that were not revealed in the original comparison. Our findings suggest that in some cases the lumbar CSF pressure was reduced by acetazolamide, as is expected [50], and that craniospinal compliance increased accordingly. As discussed above, the power of slow waves reflects both the degree of occurrence and the magnitude of these waves. The trend regarding slow waves could reflect improved craniospinal compliance with treatment, resulting in reduced wave amplitudes. However, it is also possible that in subjects treated with acetazolamide there was an decrease in the occurrence of slow waves, which is in line with a previous study that observed reduced slow wave activity in cerebral blood flow velocity after administration of acetazolamide [35]. This finding and its implications could be of interest for further research, particularly in relation to cerebral blood flow and autoregulation.

### Psychiatric comorbidities

Previously, we have reported that psychiatric disorders are very common in IIH patients compared to general population [40]. In this cohort of IIH patients who had undergone CELDA investigation, almost half of them had pre-existing psychiatric diagnosis. The most common psychiatric diagnosis was MDD. We found no differences in the baseline characteristics between IIH patients with or without psychiatric comorbidity, however the IIH patients with psychiatric comorbidity had significantly worse outcome as compared to patients without such history as shown in our previous study [40]. Comparisons between IIH subjects with or without psychiatric comorbidity showed higher lumbar CSF pressure for subjects with psychiatric comorbidity, as well as trends toward higher power of respiratory waves and lower RPPC.

### Strengths and weaknesses

Our retrospective analysis is limited by factors inherent to a secondary analysis of retrospectively collected data as well as the small sample size. Nevertheless, this issue is addressed in limited amount of previous data, as our systematic literature review shows. This study represents a detailed investigation, including continuous lumbar CSF pressure measurements, based on a well-defined cohort, which ensures a clinically comprehensive analysis and long follow-up times for these patients, which we regard as strengths of this study.

The major concern of this study is the risk for circular reasoning with the definition of symptomatic controls. Somewhat surprisingly some of these patients had also slight papilledema without visual field defects. Perhaps, this could represent early stage of IIH patients who have part of the time lumbar CSF pressure above 25 cm H_2_O. The cutoff point for lumbar CSF pressure might not unambiguously separate IIH patients from symptomatic controls, especially in patients with increased AMP and decreased craniospinal compliance.

Henceforth, the definition of symptomatic control is somewhat controversial. To overcome this problem, in the future, patients with suspected IIH and borderline lumbar CSF pressure, might benefit from control lumbar CSF pressure measurement.

Further studies, focusing on the role of CSF dynamics in patients with IIH in larger patient cohorts are needed. In 2017, we initiated a prospective collaborative multicentre study on IIH (www.iih.fi). In this study, we prospectively perform continuous lumbar CSF pressure measurement, collect biological samples for further metabolomics and genetic studies to advance understanding of the complex pathophysiology of the IIH disease, in addition to gathering all clinical variables and administrating validated questionnaires to screen and follow-up on mental well-being of patients with IIH, quality of life, and impact of their possible residual symptoms.

## Conclusion

This study confirmed that mean lumbar CSF pressure is increased in verified IIH patients compared to symptomatic controls, and also revealed that both fast and slow dynamic changes in lumbar CSF pressure were increased in IIH cases, indicating a reduced craniospinal compliance. Furthermore, we confirmed that psychiatric comorbidity is common in IIH, and found that lumbar CSF pressure was higher in cases with comorbidity than those without. Changes in some dynamic lumbar CSF pressure parameters in true IIH, such as RAP and SLOW, may be obscured by prescribed acetazolamide, which may advocate CELDA-measures before medication in feasible cases.

## Declarations

The results presented in this report are partially based on the Advanced Studies in Medicine thesis of one of the authors; Erik Martoma, “Intracranial pressure and cerebrospinal fluid dynamics in idiopathic intracranial hypertension”.
